# Experimental Investigations of Timber Beams with Stop-Splayed Scarf Carpentry Joints

**DOI:** 10.3390/ma13061435

**Published:** 2020-03-21

**Authors:** Anna Karolak, Jerzy Jasieńko, Tomasz Nowak, Krzysztof Raszczuk

**Affiliations:** Faculty of Civil Engineering, Wroclaw University of Science and Technology, Wybrzeze Wyspianskiego 27, 50-370 Wrocław, Poland; jerzy.jasienko@pwr.edu.pl (J.J.); tomasz.nowak@pwr.edu.pl (T.N.); krzysztof.raszczuk@pwr.edu.pl (K.R.)

**Keywords:** carpentry joints, scarf and splice joints, stop-splayed scarf joints (‘bolt of lightning’), static behaviour, experimental research

## Abstract

The paper presents the results of an experimental investigation of stop-splayed scarf joints, which was carried out as part of a research programme at the Wroclaw University of Science and Technology. A brief description and the characteristics of scarf and splice joints appearing in historical buildings are provided, with special reference to stop-splayed scarf joints (so-called ‘Bolt of lightning’) which were widely used, for example, in Italian renaissance architecture. Analyses and studies of scarf and splice joints in bent elements presented in the literature are reviewed, along with selected examples of analyses and research on tensile joints. It is worth noting that the authors in practically all the cited literature draw attention to the need for further research in this area. Next, the results of the authors’ own research on beams with stop-splayed scarf joints, strengthened using various methods, e.g., by means of drawbolts (metal screws), steel clamps and steel clamps with wooden pegs, which were subjected to four-point bending tests are presented. Load-deflection plots were obtained for load-bearing to bending of each beam in relation to the load-bearing of a continuous reference beam. A comparative analysis of the results obtained for each beam series is presented, along with conclusions and directions for further research.

## 1. Introduction

Wood has been used for centuries in different buildings and structures, from residential buildings, through sacral architecture, to defensive structures for settlements and towns or complex engineered structures [1]. A large number of these structures, which have survived tens or hundreds of years, now require interventions to maintain or improve their technical condition. A key factor in their development is related to joints, which enabled building elements to be connected into a single whole and for loading to be shared between elements. Historical joints pay testimony to the highly developed techniques and craftsmanship of builders at that time [2]. It should also be noted that, to date, there are few reliable references that include detailed specifications related to forming carpentry joints [3], as their creators relied mainly on their own experience and tradition.

As a rule, the behaviour of joints has a large influence on the global outcome of the structure as a whole, especially with respect to internal forces. This is why carrying out a detailed analysis of the structure as a whole requires analyses of the behaviour of joints. A simple example serves to demonstrate the role played by correctly formed scarf and splice joints connecting construction elements. In the case of vertical loading, an element with a splice joint formed on an horizontal surface can transfer the bending moment up to a quarter of the moment transferred in a continuous beam, whereas the same joint formed on a vertical surface, with the same load, can transfer a moment of half the value of the moment transferred in the continuous beam. Analyses presented in the literature [4] suggest that in the case of a nibbed scarf joint, which is the most effective solution for transferring shearing stress [3], the moment that can be transferred is only one-third of the load in the continuous beam.

To a large degree, it is the technical condition of joints that shapes the ability of a structure to transfer loading, its static behaviour and deformations. Over time, carpentry joints in historic wooden structures are worn down or even completely destroyed by loading and other external factors, which can present serious danger. The damage or destruction of a joint can threaten the safety of the whole structure due to a considerable weakening of segments where elements are connected to one another. Understanding the static behaviour of joints in wooden structures allows for a detailed and comprehensive analysis of the whole system and helps in making decisions concerning corrective measures or reconstruction work, which is acceptable from the point of view of conservation doctrine [5,6].

**Figure 1:**
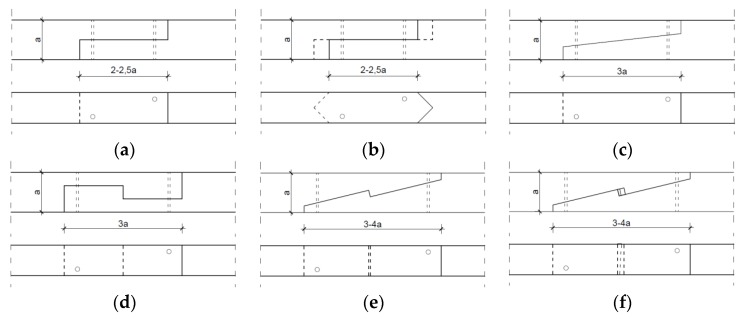
Various forms of scarf and splice joints: (**a**) splice joint, (**b**) nibbed splice joint, (**c**) nibbed scarf joint, (**d**) tabled splice joint, (**e**) stop-splayed scarf joint (‘Bolt of lightning’), (**f**) stop-splayed and tabled scarf joint with key.

Researchers distinguish many different types of carpentry joints in historic buildings, depending on their function and form (inter alia [7]). One group includes scarf joints and splice joints which enable a connection along the length of two elements. These were applied when the available material could not cover the whole beam length. Typically, whenever possible, the joints were applied in the least stressed sections, as the connecting elements at the point of contact could not bear a load greater than that in continuous sections [8]. Aside from extending foundation beams and capping beams in the building frames of historical structures, scarf and splice joints were used to extend roof frame elements, such as purlins or rafter beams. Until the introduction of glulam wood, this was a universal method for extending wood elements [9]. Scarf and splice carpentry joints are used today to restore historical joints or replenish material in historical elements of special value [10]. Examples of scarf and splice joints found in historical structures are presented in Figure 1.

The type of joint applied in a given connection is related to the function to be performed or the type of loading to be transferred. For example, a nibbed scarf joint (Figure 1c) would be used where the connection is stressed vertically and located near a support. In such a situation, the joint is responsible for transferring shear stress. In situations where tensile elements are connected, such as rafter beams, a different type of joint needs to be applied. For tensile elements, stop-splayed and tabled scarf joints with a key (Figure 1f) were used.

## 2. Stop-Splayed Scarf Joints (‘Bolt of Lightning’)

Stop-splayed scarf joints (presented inter alia in [11,12,13,14,15] and referred to as ‘Bolt of lightning’, ‘Trait-de-Jupiter’) were widely used in historical buildings. They constituted a sophisticated type of connection along the length of elements in the form of a scarf joint. In historical structures, there are also elements connected along their whole length with a so-called stop-splayed scarf joint or a composite beam (built-up beams, composite beams with a teethed joint), described inter alia in [16,17,18].

**Figure 2:**
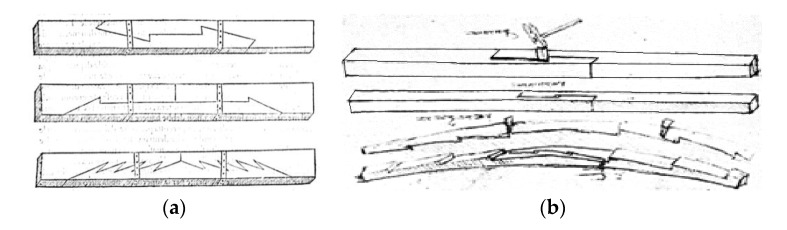
Sketches presenting scarf and splice joints in wooden beams (**a**) according to Leon Battista Alberti, (**b**) according to Leonardo da Vinci.

**Figure 3:**
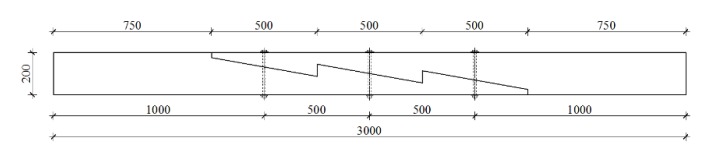
Sketch of a stop-splayed scarf joint (‘Bolt of lightning’) with dimensions of an actual element, drawing based on data from [11].

**Figure 4:**
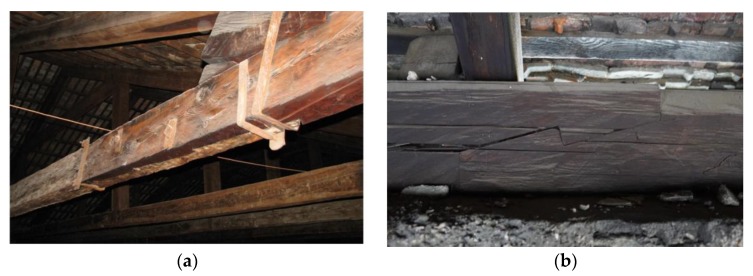
Examples of scarf and splice joining of wooden beams in existing buildings: (**a**) building in Italy, (**b**) building in Poland—13th century Czocha Castle in Sucha.

Stop-splayed scarf joints (‘Bolt of lightning’) were used in ancient times, for example, in the Roman construction of bridges, and later, in roof beam constructions and wooden ceiling construction elements right up to the end of the 19th century. A special period of development for this method of joining beam elements was that of the Italian Renaissance, which was the time of such masters as Leon Battista Alberti (Figure 2a) [19] and Leonardo da Vinci (Figure 2b) [16]. This method of connecting elements along their length was applied especially in elements subjected to tensile and bending forces. Typically, the joints were wedged, which was supposed to help in transferring loading and to ensure a tight fitting joint. Today, they are used mainly to strengthen and repair historical structures. Examples of stop-splayed scarf joints (‘Bolt of lightning’) and so-called composite beams are presented below (Figure 2, Figure 3 and Figure 4).

The few available descriptions of research concerning typical stop-splayed scarf joints (‘Bolt of lightning’) are presented in [16,17,18,20,21,22,23].

**Figure 5:**
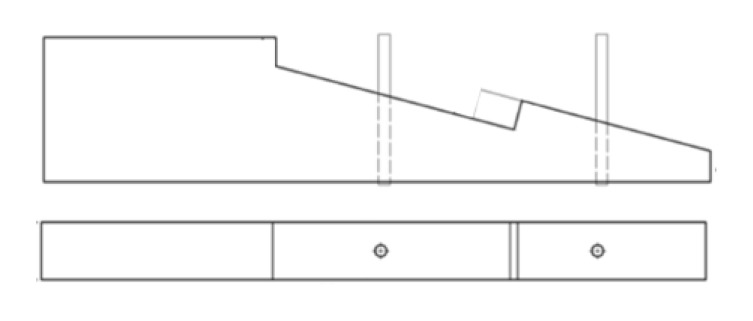
Geometry of a stop-splayed scarf joint (‘Bolt of lightning’).

An analysis of the behaviour of a stop-splayed scarf joint subjected to tensile forces is presented in Figure 5 (drawing based on data from [20]). Different methods of strengthening the joints using timber pegs and steel pins were also analysed.

An increase in estimated stiffness for the joints with strengthening was noted: 41% for the joint strengthened with wooden pegs and 52% for the joint strengthened with steel pins in relation to joint without strengthening was noted. Load-deflection plots were prepared and failure modes were described. Attention was paid to the difference in the static behaviour of the joints in relation to the material used for strengthening (wood, metal).

**Figure 6:**
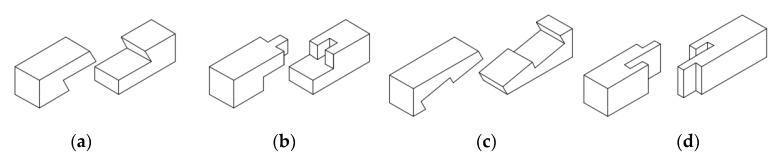
Scarf joints analysed in research: (**a**) under-squinted butt in halved scarf with two pegs, (**b**) side-halved and bridled with two pegs, (**c**) stop-splayed and tabled scarf with key and four pegs, (**d**) and face-halved and bridled scarf with four pegs.

The results of research carried out at the University of Bath in the UK by a team consisting of Walker, Harris, Hirst and others on the static behaviour of scarf joints, inter alia stop-splayed scarf joints, which are universally found in historical buildings across England, are presented in [21]. The joints studied were under-squinted butt in halved scarf with two pegs, side-halved and bridled with two pegs, stop-splayed and tabled scarf with key and four pegs, and face-halved and bridled scarf with four pegs (Figure 6) (drawing based on data from [21]).

Experimental testing [21] was carried out on model beams of 2.5 m in length joined by means of the joints listed above and 1.5 m continuous beams in order to compare results. The elements were subjected to a four-point vertical bending test and lateral bending test to provide for pure bending. Load-deflection plots were determined, and on the basis of a comparison of results for the different joint types and also the parameters for the continuous element, it was possible to determine the performance factor describing the relationship of loading and stiffness of the composite beam to that of the continuous beam. The stop-splayed scarf joint (‘Bolt of lightning’) displayed the greatest stiffness and load-bearing (28% in relation to the continuous beam) with bending in the vertical plane.

For the case of bending of the elements joined along their length with the stop-splayed scarf joint (‘Bolt of lightning’), i.e., for the so called composite beams (or built-up beams), analyses were presented by Mirabella-Roberti and Bondanelli in [16]. Based on a numerical analysis, the authors identified the most probable locations of stress concentrations, especially in the vicinity of joining planes.

**Figure 7:**
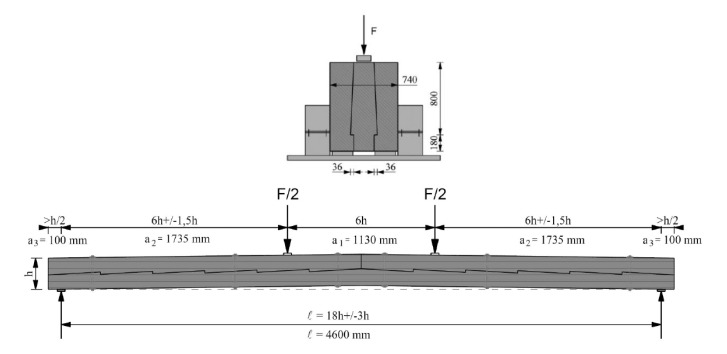
Model of a composite beam connected with a stop-splayed scarf joint and models used for testing of the joint itself and the element as a whole, image adapted from [17] with permission.

Rug et al. [17,18] present principles for developing and gauging the beams described above, which have been presented in the literature up to the 1970s. Today, it is difficult to find any principles which can provide a basis for constructing or restoring such building elements. For this reason, the University of Eberswalde in Germany carried out experimental research aimed at determining the load-bearing capacity of such elements, describing their static behaviour in terms of displacement resulting from applying loading. The research [17,18] was carried out on models constructed at a 1:1 technical scale (the dimensions adopted were the same as those in an existing rafter beam roof in a tower of a German church) and also on 1:2 scale models (Figure 7). Bending tests were carried out (in accordance with EN408), achieving an average loading at the level of approximately 57 kN; the deflection plot and flexibility modulus of the joint in the technical scale were determined in accordance with EN26891.

**Figure 8:**
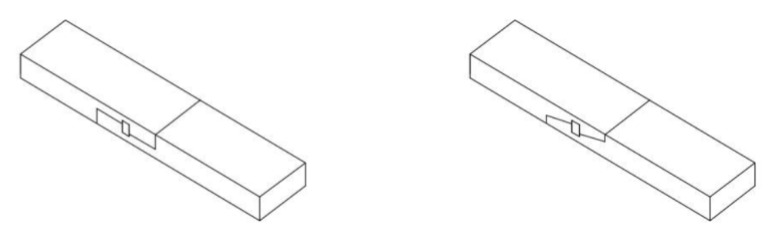
Models representing the geometry of scarf joints.

Sangree and Schafer [22,23] presented their research and numerical analysis carried out in Baltimore, USA on scarf joints with key found in traditional wooden constructions, e.g., in the Morgan Bridge, which was the subject of their analysis. They tested halved and tabled scarf joints and stop-splayed scarf joints with key (Figure 8 drawings based on data from [22,23]).

The joints were analysed as elements operating under complex loading conditions, i.e., tensile bending. In the case of the stop-splayed scarf joints with key [23], it was determined that the orientation of the key had the greatest influence on the static behaviour of the joint, as it causes a vertical pressure to the grain. In addition, special attention was paid to the presence of drawbolts as essential for sustaining the joint. In such cases, it was possible to obtain shear failure parallel to the grain, which made it possible to withstand higher stress levels.

The research presented to date in the literature concerning the static behaviour of carpentry joints has focused mainly on tenon and notched joints. There has been decidedly less research on joints typically subjected to bending (or bending and tensile stress or bending and compressive stress). It is worth noting that practically all researchers who have been concerned with this topic (in Germany inter alia Rug [17,18], in the UK Hirst et al. [21], and also in The Czech Republic Kunecky et al. [24,25,26,27,28,29] and Fajman et al. in [30,31,32,33,34,35]) underscore that there is a shortage of research for appropriately describing the static behaviour of such joints, and so proposing the most beneficial methods for repairing or strengthening them.

As a consequence (as part of a research project financed by the National Science Centre), experimental testing was carried out on the static behaviour of stop-splayed scarf joints subjected to bending. The goal was to determine the load-bearing capacity and stiffness of the joint subjected to testing and to determine the dependency of the type of joint and the method of sustaining it and its load-bearing capacity.

## 3. Experimental Tests

### 3.1. Static Schemes and Investigations Procedure

For the purposes of experimental testing, technical scale beam models were made from pine wood (Pinus sylvestris L.) of 360 cm in length and with a cross-section measuring 12 cm × 18 cm. Testing involved four series, each with three models. Series A included continuous beams as references, whereas series E, F, and G included beams with stop-splayed scarf joints in the horizontal plane. The series with joints differed from one another in terms of the methods uses to strengthen the joint, i.e.:

- series E—beams with stop-splayed scarf joints and two drawbolts (double-sided tooth plate connectors type C10 (Geka) + M12 screws);
- series F—beams with stop-splayed scarf joints and wooden keys made from oak wood and additional steel clamps;
- series G—beams with stop-splayed scarf joints and flat steel clamps and steel tie-rods.

**Figure 9:**
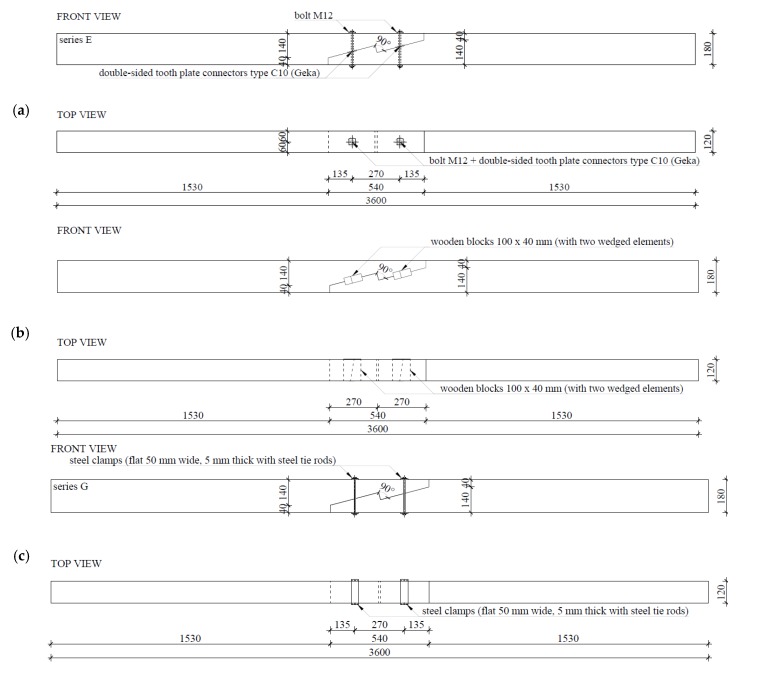
Schemes of the experimental testing models: (**a**) series E—with 2 drawbolts (double-sided tooth plate connectors type C10 + M10 screws), (**b**) series F—with wood inserts (and additional steel clamps), (**c**) series G—with steel clamps.

**Figure 10:**
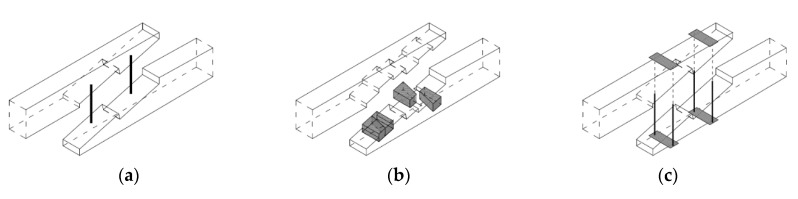
Axiometric schematics of joints used in the experimental models: (**a**) series E—with 2 drawbolts (double-sided tooth plate connectors type C10 + M10 screws), (**b**) series F—with wood inserts (and additional steel clamps), (**c**) series G—with steel clamps.

**Figure 11:**
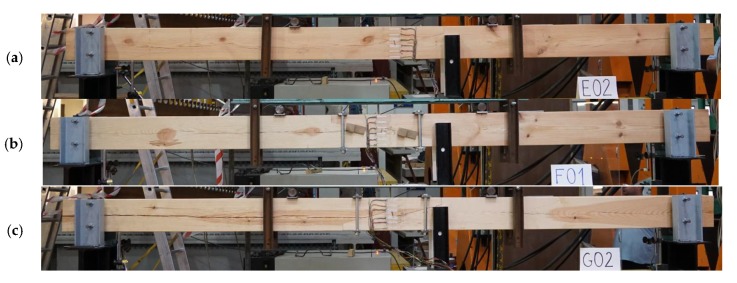
View of an example beam in each series: (**a**) series E—with 2 drawbolts (double-sided tooth plate connectors type C10 + M10 screws), (**b**) series F—with wood inserts (and additional steel clamps), (**c**) series G—with steel clamps.

With respect to the geometry of the joints, these were based on data obtained for real structures and on data from the literature. The schematics and views of the various models used for testing are presented in Figure 9, Figure 10 and Figure 11.

**Figure 12:**
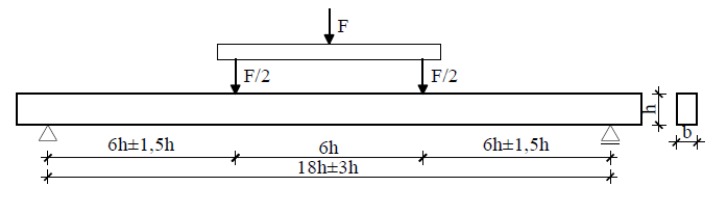
Scheme of the four-point bending test in accordance with standard procedure.

In order to determine the load-bearing and the load-deflection plot, the beam was subjected to four-point bending tests, in accordance with the standard procedure described in [36]. A schematic of the experimental testing is provided in Figure 12 (drawing based on data from [36]).

The experimental testing was carried out at the Building Construction Laboratory of the Faculty of Civil Engineering of the Wroclaw University of Science and Technology. An electronically-controlled linear hydraulic jack, the Instron 500 (Instron®, Norwood, MA, USA), was used. The results were registered using the MGC plus measurement system made by the Hottinger Baldwin Messtechnik (HBK GmbH, Darmstadt, Germany). The measurement equipment used in the experimental testing was calibrated to at least class 1 accuracy.

**Figure 13:**
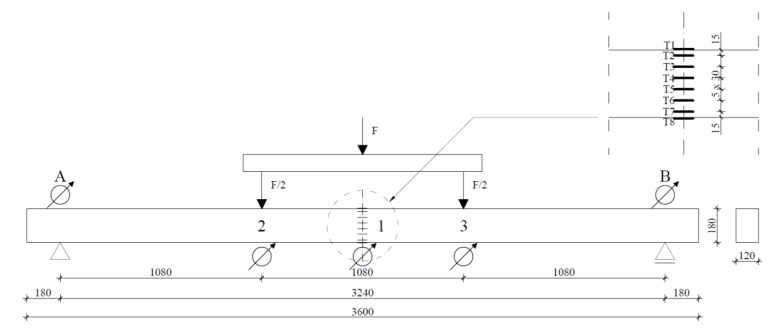
Scheme of the experimental testing site showing locations of the strain gauges.

**Figure 14:**
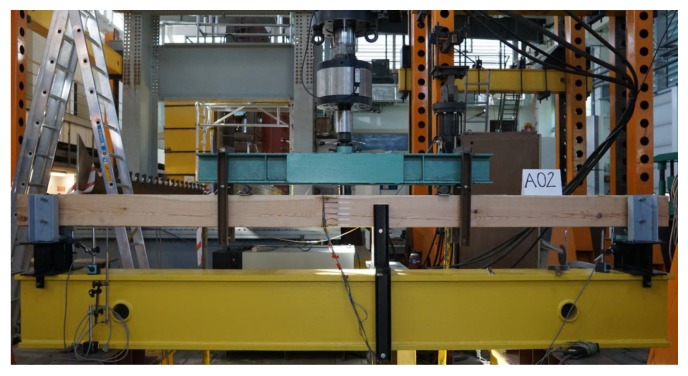
View of experimental stand for testing with an example beam.

The beams were freely supported at both ends. The span between the axes of the supports was 3.24 m. The supports included a fork support, which ensured that there was no loss in flexural static (lateral buckling). The beams were loaded symmetrically with a loading force applied at two points, thanks to which pure bending was obtained in the central part of the element. The speed of application of the loading was 5 mm/min. A schematic and view of the testing stand is presented in Figure 13 and Figure 14. Registration of the strains observed in the materials was carried out by means of a series of RL 300/50 strain gauge.

Additionally, during the course of the testing, the wood moisture was determined using a resistance hygrometer (FMW moisture meter) to take measurements in several locations on each tested beam. The moisture content of the elements was kept close to the required standard of 12% [36,37].

### 3.2. Results

**Figure 15:**
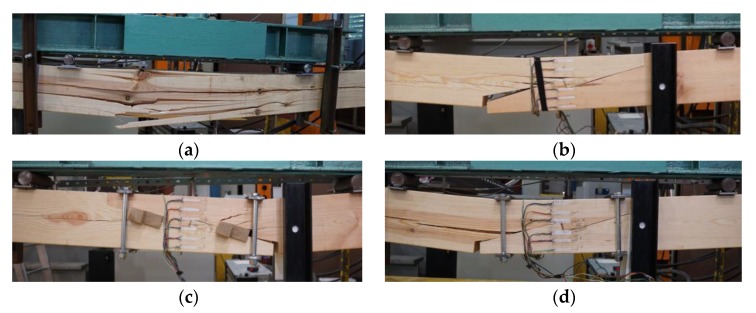
Failure views for selected beams (**a**) A01, (**b**) E03, (**c**) F01, (**d**) G02.

All the beam models (altogether 12 elements) were loaded at a constant speed right up to their failure. The failure of beams in series A resulted from the shearing of fibres in the bottom part of the central area of the beam where earlier there were no visible cracks (except for cracks in the vicinity of knots in the beams). Sudden failure with no prior signs occurred. Generally, the failure of beams in series E, F, and G involved a loosening of the joint in the lower zone of the central part of the beam and the appearance of cracks and fractures on the edges of the joint and in points weakened as a result of earlier flaws (such as knots or primary cracks of the beam). The tested joint was unsymmetrical. In the right part of the joint, the beam elements were bent and pressed to each other. In the left part of the joint, the elements were also bent, but the bottom element was not pressed and the joint was loosened. Destruction occurred in the left part of the joint by breaking the upper element (visible cracks above the edge of the joint). The cross-section of the destruction was in the joint area. Failure resulted from delamination due to stretching across the fibres. The failure views of selected experimental models are presented in Figure 15.

Table 1 presents the value of the ultimate forces F_u_ obtained for each of the beam series. Ratio of mean destruction force for specified series to reference beam series is F_uX_/F_uA_, where X stands for E, F, or G (and expresses mean destructive force for this series).

**Figure 16:**
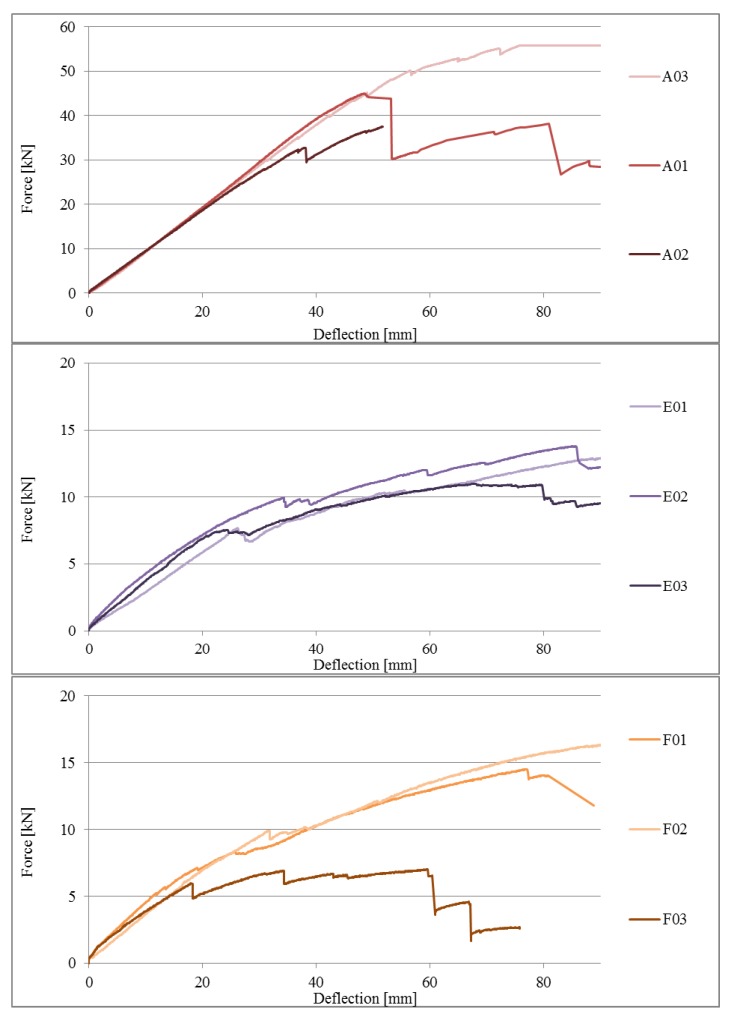

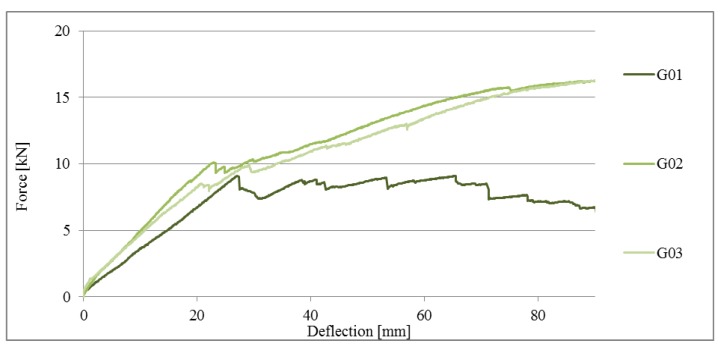
Load-deflection plots for beams in series A, E, F, and G.

Load-deflection plots (in the central part of the beam span, i.e., in point 1) for beams in the various series are presented in Figure 16.

**Figure 17:**
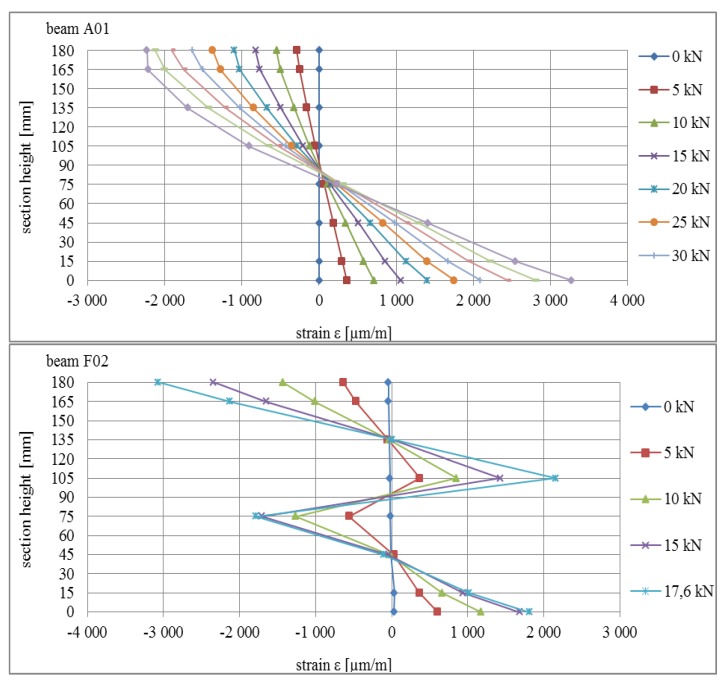

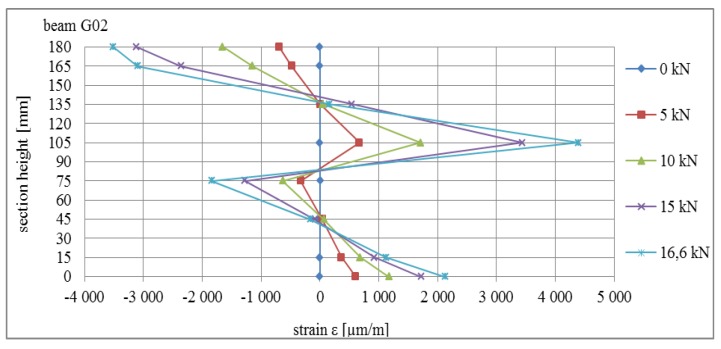
Strain profile in the cross-section for selected beams subjected to bending.

Deformations in the area of point 1 in the central part of the beams are presented in Figure 17. The deformation profiles are consistent with expected curves: for beam A01, the standard image of deformation in the cross-section for the continuous beam was obtained, whereas for beams E02, F02, and G02, it was noted that the typical curve shape for the composite cross-section was as presented inter alia in [18].

### 3.3. Analysis of Results

**Figure 18:**
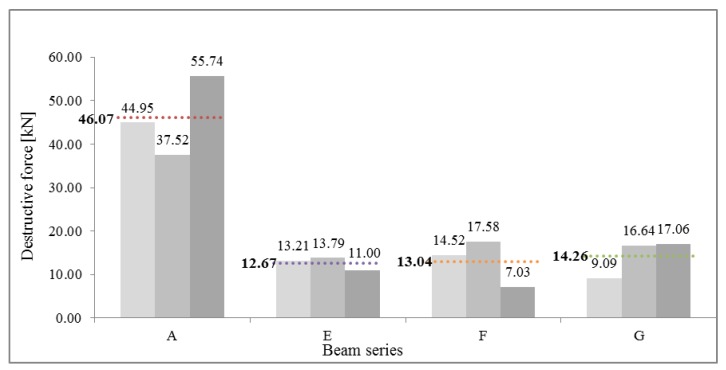
Comparison of values obtained for ultimate force values for beams of series A, E, F, and G.

The mean ultimate force value for beams in series A (continuous beams) was 46.07 kN. The value of the mean load bearing for bending for this series was 24.88 kNm. Compared to the reference beams, beams joined with stop-splayed scarf joints, i.e., beams in series E, F and G, achieved lower load-bearing levels, which were comparable to one another. The highest values were obtained for series G beams with a mean ultimate force of 14.26 kN (load-bearing for bending of 7.70 kNm), which constituted 31.0% load-bearing in relation to reference beams. The largest variation in results was obtained for this series (variation coefficient exceeds 30%). Beams of series E and F obtained lower ultimate force values: series E—12.67 kN (load-bearing for bending 6.86 kNm), series F—13.04 kN (load bearing for bending 7.04 kNm) which constitutes respectively 27.5% and 28.3% load-bearing in relation to the reference beam. The results of series E were characterised by the smallest variation with an indicator of not much more than 10%. A comparison of the values of ultimate force values for beams in series A, E, F, and G is presented in Table 2 and in the curve in Figure 18.

**Figure 19:**
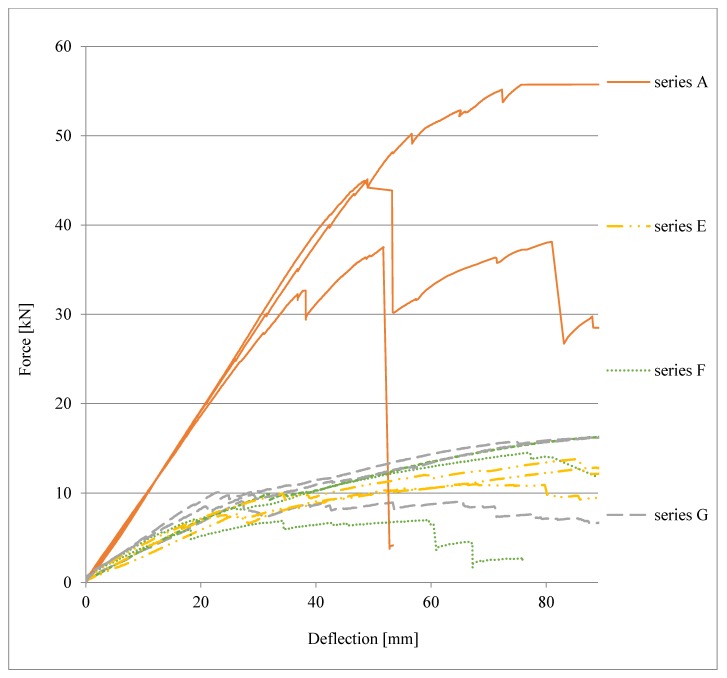
Comparison of load-deflection plots of beams of series E, F, G to the reference beam of series A.

As part of the comparative analysis, in Figure 19, a comparison of the load-deflection plots of the beams of series E, F, and G to the reference beams of series A is presented. It should be noted that the tested beam series attained similar values for final deflection, but with different levels of force, which are several times higher for series A, whereas the values for beams of series E, F, and G were similar to one another.

An analysis of the graph (curves for series E, F, and G) shows a change in the nature of the static behaviour of the joint when strain η is equal to approximately 60% of the elastic to ductile state. Estimations on the basis of the load-deflection plots of the ‘stiffness parameter’ in the elastic state, calculated as the ratio of loading force value to deflection (1) for the beams representing series A, E, F, and G, are as follows: for A01—0.94 kN/mm, for E01—0.31 kN/mm, for F01—0.32 kN/mm, and for G02—0.45 kN/mm.

(1) $$k=\mathrm{tg}\alpha =\frac{F_{i}}{u_{i}}\left[\mathrm{kN}/\mathrm{mm}\right]$$

It should be noted that the parameters for beam E01 and F01 are close to one another and amount to approximately 30% of the stiffness parameter for the continuous beam A01, whereas in the case of beam G02, the parameter amounts to approximately 50% of the reference value. The most important test results obtained for the beams from the specified series in relation to the reference beam (continuous beam) are presented in Table 3.

## 4. Conclusions

Stop-splayed scarf joints are common in historical structures where there are elements that are subject to bending, tensile stress, and bending with tensile stress, which are to be found primarily in roof framing elements, but also less commonly in wooden ceilings. When appropriately strengthened, e.g., with steel clamps or screws, etc., these joints can transfer bending loads. These types of strengthened joints have not been described to date in the literature.

The obtained test results can be helpful in the design and strengthening of this type of joint, especially in historical buildings.

Experimental testing carried out on technical-scale models presented the static behaviour of such connections subjected to bending load compared to bending of a continuous reference beam. The bending load-bearing levels obtained for beams with joints compared to the reference beam amounted to approximately 30% (27.5–31% depending on the type of strengthening method used). The stiffness levels obtained in laboratory experimental testing amounted to 30–40% in comparison to the reference beam. The lowest load-bearing capacity and stiffness were obtained for beams in series E, which were strengthened with drawbolts, and higher values for beams strengthened with steel clamps and wooden pegs.

Despite a small sample from a statistical point of view, the obtained test results confirm the results of other researchers regarding the level of load-bearing capacities observed in beams with joints of a similar type.

It is important to underscore that the laboratory testing involved three models in each series, which resulted in large variations in results in some series (as, for example, with the beams in series F, where the variation coefficient for ultimate force amounted to approximately 40%). It can be concluded that the high variation coefficient obtained resulted from primary flaws in the material, and not from the behaviour of the joints themselves. As a natural material, wood is characterised by large variations due to the presence of knots, cracks etc.

The experimental investigation that was carried out allowed us to obtain a description of the static behaviour of stop-splay scarf joints subjected to bending. For more precise analyses and conclusions, numerical analyses and further laboratory testing are recommended. Given the failure mechanisms observed and the deformations in the lower edges of the joint, it is recommended that in further experimental testing, consideration is given to testing asymmetrical joint strengthening, with greater strengthening in the zone, where the largest deformations were obtained. 

As a next step of the research programme, numerical analyses and further experimental investigations in the laboratory of beams with different types of joints will be carried out on the described joints. The main goal of this research is to determine the solution of optimizing the design of carpentry joints in bent wooden elements.

## Figures and Tables

**Table 1:** Ultimate forces for beams in each series.

		Beam Series			
		A	E	F	G
Ultimate force Fu	[kN]	44.95	13.21	14.52	9.09
		37.52	13.79	17.58	16.64
		55.74	11.00	7.03	17.06
Mean ultimate force for each beam series $\bar{F_{u}}$	[kN]	46.07	12.67	13.04	14.26
Standard deviation s	[kN]	9.16	1.47	5.43	4.49
Variation coefficient ν	[%]	19.89	11.62	41.61	31.45
Ratio of mean destruction force for specified series to reference beam series		1.00	0.27	0.28	0.31

**Table 2:** Comparison of load-bearing for beams in the various series.

		Beam Series			
		A	E	F	G
Mean ultimate force for each beam series $\bar{F_{u}}$	[kN]	46.07	12.67	13.04	14.26
Mean load-bearing for bending for each beams series $\bar{M_{R}}$	[kNm]	24.88	6.84	7.04	7.70
Ratio of load-bearing for specified series to reference beam series		1.00	0.27	0.28	0.31

**Table 3:** Presentation of results for beams from specified series.

	Beam Series			
	A	E	F	G
Beam Type (Strengthening of the Joint)	Reference-Continuous Beam	Drawbolts	Wooden Pegs (keys)	Steel Clamps
Mean ultimate force $\bar{F_{u}}$ [kN]	46.07	12.67	13.04	14.26
Mean load-bearing in bending $\bar{M_{R}}$ [kNm]	24.88	6.84	7.04	7.70
Ratio of load-bearing as compared to reference beam series	1.00	0.27	0.28	0.31
Mean deflection u1 in the middle of the span with a load of 10 kN [mm]	12.34	37.44	32.48	24.59
Ratio of deflection as compared to reference beam series	1.00	3.03	2.63	1.99
Mean stiffness parameter k [kN/mm]	0.94	0.29	0.32	0.39
Stiffness parameter as compared to the reference beam series	1.00	0.32	0.35	0.43
